# The management of menopause in women with a history of endometriosis: a systematic review

**DOI:** 10.1093/humupd/dmx011

**Published:** 2017-05-11

**Authors:** L.C. Gemmell, K.E. Webster, S. Kirtley, K. Vincent, K.T. Zondervan, C.M. Becker

**Affiliations:** 1 Case Western Reserve School of Medicine, 10900 Euclid Avenue, Cleveland, OH 44106, USA; 2 Endometriosis CaRe Centre, Nuffield Department of Obstetrics and Gynaecology, University of Oxford, John Radcliffe Hospital, Women's Centre, Oxford OX3 9DU, UK; 3 Centre for Statistics in Medicine, Nuffield Department of Orthopaedics, Rheumatology and Musculoskeletal Sciences, Botnar Research Centre, Windmill Road, Oxford OX3 7LD, UK; 4 Wellcome Trust Centre for Human Genetics, University of Oxford, Roosevelt Drive, Oxford OX3 7BN, UK

**Keywords:** endometriosis, menopause, HRT, unopposed oestrogen, combined HRT, tibolone, recurrence, malignant transformation

## Abstract

**BACKGROUND:**

Endometriosis is typically regarded as a premenopausal disease, resolving after natural or iatrogenic menopause due to declining oestrogen levels. Nonetheless, case reports over the years have highlighted the incidence of recurrent postmenopausal endometriosis. It is now clear that both recurrence and malignant transformation of endometriotic foci can occur in the postmenopausal period. Postmenopausal women are commonly treated with hormone replacement therapy (HRT) to treat climacteric symptoms and prevent bone loss; however, HRT may reactivate endometriosis and stimulate malignant transformation in women with a history of endometriosis. Given the uncertain risks of initiating HRT, it is difficult to determine the best menopausal management for this group of women.

**OBJECTIVE AND RATIONAL:**

The aim of this study was to systematically review the existing literature on management of menopausal symptoms in women with a history of endometriosis. We also aimed to evaluate the published literature on the risks associated with HRT in these women, and details regarding optimal formulations and timing (i.e. initiation and duration) of HRT.

**SEARCH METHODS:**

Four electronic databases (MEDLINE via OVID, Embase via OVID, PsycINFO via OVID and CINAHL via EbscoHost) were searched from database inception until June 2016, using a combination of relevant controlled vocabulary terms and free-text terms related to ‘menopause’ and ‘endometriosis’. Inclusion criteria were: menopausal women with a history of endometriosis and menopausal treatment including HRT or other preparations. Case reports/series, observational studies and clinical trials were included. Narrative review articles, organizational guidelines and conference abstracts were excluded, as were studies that did not report on any form of menopausal management. Articles were assessed for risk of bias and quality using GRADE criteria.

**OUTCOMES:**

We present a synthesis of the existing case reports of endometriosis recurrence or malignant transformation in women undergoing treatment for menopausal symptoms. We highlight common presenting symptoms, potential risk factors and outcomes amongst the studies. Sparse high-quality evidence was identified, with few observational studies and only two randomized controlled trials. Given this paucity of data, no definitive conclusions can be drawn concerning risk.

**WIDER IMPLICATIONS:**

Due to the lack of high-quality studies, it remains unclear how to advise women with a history of endometriosis regarding the management of menopausal symptoms. The absolute risk of disease recurrence and malignant transformation cannot be quantified, and the impact of HRT use on these outcomes is not known. Multicentre randomized trials or large observational studies are urgently needed to inform clinicians and patients alike.

## Introduction

### Endometriosis and oestrogen dependence

Endometriosis is a disease that affects an estimated 6–10% of reproductive aged women, totalling approximately 176 million women worldwide (Bulun, 2009). It is defined as the presence of endometrial-like tissue in extrauterine locations and is a chronic condition associated with debilitating pelvic pain, dyspareunia, dysuria, dysmenorrhoea and infertility. However, due to a lack of reliable diagnostic tools and the non-specific nature of the symptoms, there exists a widely recognized delay in diagnosis of 8–10 years (Ahn *et al.*, 2017). Consequently, the economic impact is substantial, as chronic and debilitating pain from endometriosis may hinder work productivity, while infertility can cause major psychosocial and financial strain to affected women and their partners (Simoens *et al.*, 2007, 2012).

The pathophysiology of endometriosis is complex and not completely understood. Sampson's retrograde menstruation theory, which states that endometrial cells travel backwards through the fallopian tubes during menses to reach the peritoneal cavity, has gathered the most robust support (Vercellini *et al.*, 2014). Oestrogen dependence, progesterone resistance, inflammation and genetic predisposition represent some of the pathophysiological hallmarks of this disease (Burney and Giudice, 2012). The central feature is oestrogen-dependent growth. Endometriotic lesions pathologically overexpress oestrogen receptor beta (ERβ) (>100× higher expression compared to endometrial tissue) and have been demonstrated to express (i) high levels of steroidogenic acute regulatory protein (StAR) and P450 aromatase, and (ii) reduced levels of 17beta hydroxysteroid dehydrogenase Type 2. This expression profile results in locally elevated levels of the biologically active form of oestrogen (oestradiol) (Kitawaki *et al.*, 2002; Bulun *et al.*, 2012). These molecular studies are supported by clinical observations of disease regression, symptom relief and alleged ‘cures’ for endometriosis as women achieve a hypo-oestrogenic state through iatrogenic or natural menopause (Inceboz, 2015).

### Transition to menopause

Understanding the altered hormonal milieu in endometriosis has enabled clinicians to exploit oestrogen dependence in their management, prescribing medications to suppress ovarian function or alter local oestrogenic effects. However, in severely symptomatic cases, first-line medical therapy (including the oral contraceptive pill or progestogens) or laparoscopic excision of endometriotic lesions may prove insufficient, and induction of menopause via GnRH analogues or oophorectomy is indicated (Dunselman *et al.*, 2014). Surgically or medically induced menopause is associated with a swift and dramatic fall in oestrogen levels. This decline may relieve endometriosis-related symptoms, but can simultaneously trigger menopausal symptoms. These symptoms are diverse and include hot flushes, vaginal dryness, sleep and mood disturbances, night sweats and painful intercourse, among others. While these symptoms occur in many women who naturally transition into menopause, they are especially prevalent and severe in women with a sudden onset of the hypoestrogenic state (Hendrix, 2005). The gold standard for treatment of menopausal symptoms has traditionally been hormone replacement therapy (HRT). HRT has been crucial for achieving symptom relief and improving the quality of life of millions of menopausal women, although these successes have been accompanied by safety concerns regarding specific preparations and dosages (Manson *et al.*, 2013).

### Appropriateness of HRT

Many studies have explored the efficacy and safety of HRT in postmenopausal women with climacteric symptoms (Rossouw *et al.*, 2002; Rozenberg *et al.*, 2013); however, few studies have investigated the use of hormonal therapy in postmenopausal women with a history of endometriosis. Two specific concerns are present in this group of women. Firstly, there is the possibility that exogenous oestrogen will reactivate growth of endometriotic deposits and cause symptomatic recurrence. Secondly, there is a concern that oestrogen will promote malignant transformation of residual endometriotic tissue. Sampson first described malignant transformation of ovarian endometriosis in 1925 (Sampson, 1925) and although its pathogenesis is not fully understood, oxidative stress, inflammation and an altered hormonal milieu have been implicated as contributing factors (Nezhat *et al.*, 2014). Malignant transformation is thought to be a multistep pathway in which normal endometriotic tissue progresses to an atypical intermediate stage, and finally to invasive carcinoma (Gadducci *et al.*, 2014). These sequential steps towards malignancy have been associated with genetic alternations in PTEN, TP53 and ARID1A and have been demonstrated in endometriosis-associated cancers (Munksgaard and Blaakaer, 2012). In a recent animal study using a rodent model of endometriosis (adult female Sprague-Dawley rats, aged 8–12 weeks), treatment with unopposed oestrogen successfully induced malignant transformation of endometriotic foci (Wang *et al.*, 2015). Mechanistically, oestrogens affect PTEN expression in human endometrial cells and are associated with increased proliferation, direct cell damage and increased risk of acquiring somatic mutations (Turbiner *et al.*, 2008).

However, the impact of declining oestrogen levels should not be underestimated. Menopausal symptoms affect the lives of millions of women worldwide. The hypo-oestrogenic state can significantly impair the quality of life by making sexual intercourse uncomfortable or painful, causing sleep deprivation, or resulting in mood changes. Furthermore, declining systemic oestrogen levels are a risk factor for cardiovascular and bone disease (Gallagher, 2007; Rosano *et al.*, 2007). The use of HRT has been shown to reduce the risk of such conditions and improve the quality of life of symptomatic women (Langer, 2017).

The decision whether or not to prescribe HRT in general, and particularly in women with a history of endometriosis, is therefore a complex clinical decision and may also take into account other risk factors, such as residual disease after surgery (Clayton *et al.*, 1999) and obesity which causes increased aromatase activity in peripheral tissues resulting in higher systemic oestrogen levels (Zanetta *et al.*, 2000).

Our study aimed to conduct a systematic review of the literature investigating a critical question: What is the current evidence on the management of menopausal symptoms in women with a history of endometriosis? We aimed to cover the literature on a number of sub-questions, including: What are the various treatment options to manage menopausal symptoms in these women? What are the risks associated with HRT in this cohort? Should HRT be given immediately following surgically induced menopause or be delayed? What preparations are most appropriate, and for how long should treatment be given? We aimed to synthesize the literature in a comprehensive manner, and hoped to aid the design of future research in this area. Given the prevalence of endometriosis and the inevitability of eventual menopause in these women, this is clearly an important question that warrants robust, evidence-based guidelines.

## Methods

This systematic review was registered and accepted for inclusion in PROSPERO (Gemmell *et al.*, 2016) in July 2016 (PROSPERO ID number: CRD42016042024).

### Search strategy

We searched four electronic databases (MEDLINE via OVID, Embase via OVID, PsycINFO via OVID and CINAHL via EbscoHost), from database inception until 26 June 2016, using a combination of relevant controlled vocabulary terms and free-text terms searched in the title or abstract fields related to ‘menopause’ and ‘endometriosis’. No study type, language or date limits were applied to the search. An example of the search strategy used for the MEDLINE database is included in Supplementary Table S1.

### Inclusion criteria

All retrieved studies were uploaded to EndNote and duplicates were deleted. One reviewer (L.G.) sifted the full library (titles/abstracts), and two reviewers (K.W., C.B.) sifted 10% of the library (randomly selected using EndNote Record Number) to assess concordance. The full text of potentially relevant articles was retrieved to assess whether the paper should be included. Inclusion criteria were that the study population included postmenopausal women with a confirmed, or clinically suspected, history of endometriosis, and the article discussed management of menopausal symptoms. All study designs were included (case reports, observational studies and clinical trials). We excluded articles that did not discuss any form of menopausal management (e.g. HRT, tibolone or other preparations). We excluded narrative review articles and organizational guidelines in an attempt to focus the review on primary literature. Conference abstracts were also excluded.

The reviewers shared their lists of included studies, and concordance was determined. When there were disparities in the list, consensus was reached through discussions between the reviewers.

### Quality assessment

Quality of included studies was assessed independently by two reviewers (L.G., K.W.) using the GRADE criteria (Guyatt *et al.*, 2011). Assigned ratings were compared and a third reviewer (C.B.) was consulted when there were disagreements.

### Data extraction

Data were extracted into a standard form by one reviewer (L.G). For case reports, the following information was extracted: patient age at presentation, presenting symptoms, ureteral involvement (yes or no), type (surgical vs. natural) and timing (years previously) of menopause, stage and extent of endometriosis before menopause, reported menopausal symptoms, treatment provided, duration of follow-up, method of outcome assessment, outcome (recurrence, malignant transformation, side effects, mortality) and recommendation (if provided).

For all other study types, information on study design, study objective, sample size, participants’ characteristics, intervention, method of outcome assessment, outcome, duration of follow-up and recommendation (if provided) was recorded.

### Data synthesis and analysis

The nature of the evidence retrieved by our search (predominantly case reports, and a small number of heterogeneous observational studies and clinical trials) meant that meta-analysis was not possible, thus a narrative synthesis of the data is provided.

Where possible, if not reported in the original article, risk ratios (RRs) for specific outcomes were calculated using RevMan (Review Manager (RevMan) [Computer program]. Version 5.3 2014).

## Results

### Included studies

**Figure 1:**
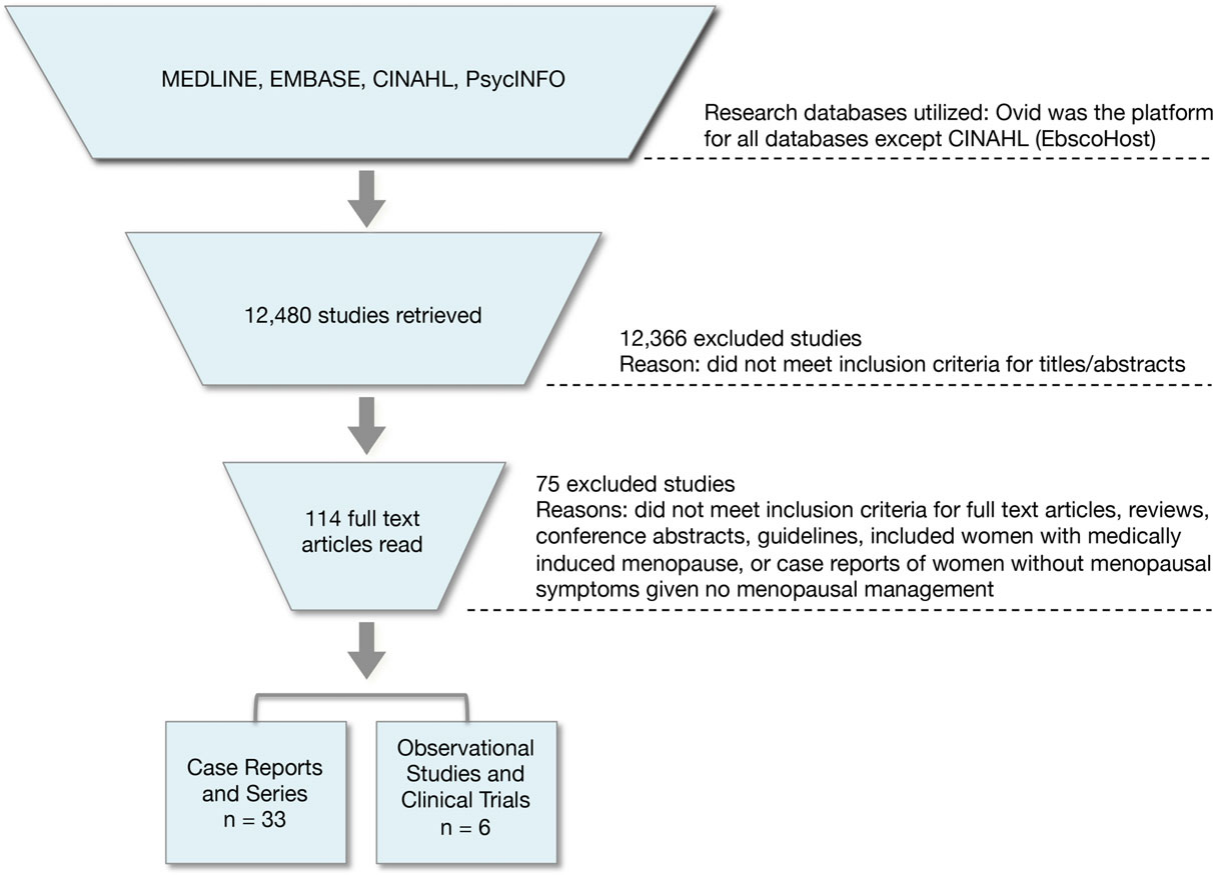
Flow diagram depicting inclusion and exclusion decisions throughout the review process.

Searches across all four databases retrieved 17 488 studies. Duplicates (5008) were removed, leaving 12 480 studies (Fig. 1). After reading titles and abstracts, 12 366 failed to meet inclusion criteria. The full-text versions of the remaining 114 studies were read in their entirety. Of these 114 studies, 75 were excluded because they did not meet our inclusion criteria. This left 39 included studies: 33 case reports and 6 observational studies and clinical trials.

The majority of relevant articles identified by the search were individual case reports or case series describing the recurrence of endometriosis or malignant transformation in postmenopausal women. A small number of observational studies and clinical trials were also identified. We first present a summary of the data from case reports and case series to give context, before describing the results of the observational cohorts and trials.

### Case reports and case series (33 studies; 48 patients)

There were 32 case reports/series including 42 patients identified by our search. An additional article describing endometriosis-related malignancies in six women who had taken oestrogen replacement was retrieved (Leiserowitz *et al.*, 2003). This article is discussed separately, as insufficient data are reported for the individual women to enable inclusion in our summary statistics.

Outcome evidence provided by these reports was assessed as very low quality given their observational nature and inherent risk of publication bias. Summary characteristics of the 42 patients are presented in Table I. The age of included patients ranged between 30 and 75 years at presentation (mean age: 52 years). Of 42 patients, 40 had prior histories of endometriosis, either (i) confirmed by intraoperative visualization and/or histologically after laparoscopic excision (*n* = 34), (ii) suspected given the presence of symptoms (infertility, pelvic pain, menorrhagia) (*n* = 2), or (iii) assessed by unspecified methodology (*n* = 4). Two patients did not have premenopausal endometriosis diagnoses, but were speculated by the case report authors to have had such and are thus included in our analysis. There were 36 patients who went through a surgically induced menopause (procedures involving oophorectomy), and four patients went through menopause naturally (one of these four was diagnosed with premature ovarian insufficiency). An additional two patients are believed to have gone through natural menopause, and underwent oophorectomy at ages 57 and 60. HRT was given as treatment for (*n* = 12) or prevention of (*n* = 30) menopausal symptoms. The mean duration of HRT use prior to presentation was 7.8 years (range: 4 months to 20 years). Of 36 patients who had undergone hysterectomy, 31 used unopposed oestrogen therapy.

**Table I:** Summary characteristics from case reports and series.

Case reports and series (GRADE (Guyatt *et al.*, 2011) scoring: very low quality)	
Number of patients (*n*)	42
Age range (years) (mean (years))	30–75 (52)
Type of menopause (*n*)	Surgical: 36 Natural: 4 Presumed natural + oophorectomy later: 2
Mean duration of HRT (years)	7.8
Unopposed oestrogen (*n*)	31
Endometriosis recurrence (*n*)	17
Malignant transformation (*n*)	25
Mortality (*n*)	3

The two main outcomes reported were endometriosis recurrence (*n* = 17) and malignant transformation (*n* = 25). For analysis, case reports and series were divided by these two main outcomes. One case series was included in both outcomes as it described three patients with endometriosis recurrence and one patient with malignant transformation (Taylor *et al.*, 1999).

### Endometriosis recurrence in women on HRT (17 patients)

Thirteen case reports and case series were identified reporting endometriosis recurrence in menopausal women given HRT for the treatment or prevention of menopausal symptoms. These included 17 patients between the ages of 30–65 (median age: 46 years) (Table II). All of the included women had undergone treatment with exogenous oestrogens in some form. Skor *et al.* (1977) was the earliest report retrieved by our search. This case was a 48-year-old Caucasian woman who presented with a 2-month history of painless haematuria and decreased urinary stream on voiding. She had undergone a total abdominal hysterectomy with bilateral salpingo-oophorectomy (TAH + BSO) with endometriosis found in the specimen and confirmed by histology. She had been prescribed conjugated oestrogens (Premarin 1.25 mg/day) following surgery and continued these for 6 years until her presentation. On physical examination, a 7 cm × 8 cm mass starting in the midline and extending to the left pelvic wall was palpated and the patient underwent cystoscopy. Postmenopausal bladder endometriosis was diagnosed histologically. Oestrogens were discontinued and intramuscular medroxyprogesterone acetate (1 gm per week) was administered for 2 months. Despite this, there was no significant alteration in the size of the mass. Shortly afterwards, due to symptom recurrence, the endometriotic lesion was removed surgically. The patient had no complaints 1-year post treatment. The authors commented that exogenous oestrogens play a role in the stimulation and development of postmenopausal endometriosis.

**Table II:** Case reports and series reporting the postmenopausal recurrence of endometriosis after HRT in women with a history of endometriosis.

Author, date (# patients)	Patient Age (years)	Presenting symptoms [ureter involvement]	Medical history and menopause	HRT [duration]	Diagnosis	Treatment [follow-up: patient status]
Skor *et al.* (1977) (*N* = 1)	48	Painless haematuria + palpable bladder mass [No]	Endometriosis Surgical menopause	Oestrogen-only HRT Conjugated oestrogen tablets [6 years]	Postmenopausal bladder endometriosis	Discontinued oestrogens + initiated Depo-Provera + surgery [1 year: no complaints]
Stewart and Ireland (1977) (*N* = 1)	65	Intermittent painless haematuria [No]	Leiomyoma, no confirmed history of endometriosis, but speculated by authors Unclear menopause (underwent TAH + right SO at age 59)	Oestrogen-only HRT Oestrogen tablets [3 years]	Bladder endometriosis extending into bowel	Surgery [6 weeks: cystoscopy revealed normal appearing bladder and bimanual examination was normal]
Kapadia *et al.* (1984) (*N* = 1)	56	Painless haematuria; two episodes of gross haematuria 10 days before admission [Yes]	Uterine leiomyomas, bilateral endometriomas, right fallopian tube endometriosis Surgical menopause	Oestrogen-only HRT Conjugated oestrogen tablets [‘long term’]	Postmenopausal ureteral endometriosis	Surgery [18 months: no complaints]
Ray *et al.* (1985) (*N* = 1)	64	Painless haematuria [Yes]	Right ovarian endometriosis Surgical menopause	Oestrogen-only HRT Conjugated oestrogen tablets [13 years]	Ureteral obstruction secondary to adenomatous hyperplasia arising in endometriosis	Surgery [Not specified]
Manyonda *et al.* (1989) (*N* = 2)	47 39	Three day history of vomiting and right iliac fossa pain [Yes] Two day history of severe left loin and left iliac fossa pain [Yes]	Bilateral ovarian endometriosis Extensive endometriosisBoth surgical menopause	Oestrogen-only HRT Oestradiol implant, ethinyloestradiol [5 years, 3 days 2 weeks prior to presentation] Oestradiol implant [9 years]	Large chocolate cyst (3 cm in diameter) compressing ureter at bifurcation of iliac vessel Chocolate cyst (3–4 cm in diameter), endometriosis in the cyst wall and end-stage obstructive uropathy of the kidney	Surgery [2 years: no complaints, on Danazol] Surgery [6 months: no complaints, on Danazol]
Goh and Hall (1992) (*N* = 1)	54	5-month history of left iliac fossa pain, especially during her monthly withdrawal bleeding [No]	Endometriosis suggested by symptoms: primary infertility and recurrent left iliac fossa pain Natural menopause	Combined HRT Conjugated oestrogen tablets + oral medroxyprogesterone [1 year]	8 cm left ovarian endometrioma adherent to sigmoid colon—uterus had extensive endometrial deposits on serosal surface	Surgery [2 years: no complaints, on Premarin to treat menopausal symptoms]
Joseph *et al.* (1994) (*N* = 1)	30	Recurrent haemoptysis and left-sided haemothorax [No]	Extensive pelvic endometriosis, pleural endometriosis Surgical menopause	Combined HRT Oestrogen and progesterone [4 months]	Recurrent thoracic endometriosis	Surgery [9 months: no complaints, advised to delay HRT]
Taylor *et al.* (1999) (*N* = 3)	42 40 38	Left abdominal pain and vaginal bleeding [Yes] Severe lower abdominal pain [No] Severe dyspareunia [No]	All had severe or extensive endometriosis All surgical menopause	Oestrogen-only HRTAll used oestradiol implants [3 years] [2 years] [2 years]	Endometriotic mass in left pelvis obstructing ureter Extensive pelvic endometriosis Endometriosis of vaginal vault	Surgery [6 years: no complaints, on continuous combined hormone replacement] Surgery + discontinued oestradiol implant [Not specified] Surgery [Not specified, on tibolone]
Badawy *et al.* (2004) (*N* = 2)	35 40	Vaginal bleeding that intensified and became constant, severe cramping [No] Worsening vaginal bleeding of several months duration [No]	Severe chronic pelvic pain, dyspareunia, dysmenorrhoea, bilateral ovarian endometriosis Severe endometriosis, right ovarian endometriosis, adenomyosis Both surgical menopause	Oestrogen-only HRT and combined HRT Oestrogen patch followed by ‘various regiments of oestrogen and progestogen’ [3 months, 1 year] Oestrogen patch [19 months]	Both—endocervical endometriosis	Surgery [Not specified] Surgery [Not specified, on ERT]
Taylor *et al.* (2005) (*N* = 1)	46	Haematuria and rectal bleeding [No]	Endometriosis Surgical menopause	Oestrogen-only HRT Oestradiol implants, followed by oestradiol patches [Not specified, but patient stopped HRT 12 months prior to presentation]	5 cm endometriotic nodule in the sigmoid that was adherent to the bladder; florid endometriosis with polypoid endometrial mucosal lesions with vascular invasion involving both bowel and bladder	Surgery [Not specified]
Chahine *et al.* (2007) (*N* = 1)	46	3 episodes of haemoptysis synchronous on 1st day of menstrual cycle [Unknown]	Endometriosis suspected by symptoms: menorrhagia Natural menopause	Combined HRT Oestradiol + cyproterone [3 years]	Catamenial haemoptysis due to endobronchial endometriosis	Terlipressin + Discontinued HRT [2 years: no complaints]
Mattar *et al.* (2008) (*N* = 1)	49	Asymptomatic pelvic mass detected during routine examination [No]	Pelvic endometriosis associated with infertility and adhesions Premature ovarian insufficiency	Combined HRT Conjugated equine oestrogens + norethisterone [7 years]	Intestinal endometriosis presenting as a pedunculated endometrioma	Tibolone + Surgery [Not specified]
Giarenis *et al.* (2009) (*N* = 1)	44	Painless vaginal bleeding for 4 weeks [Yes]	Endometriosis and infertility Surgical menopause	Oestrogen-only HRT Conjugated oestrogen [10 years]	Endometriotic mass (8 × 7 × 6.5) involving the vaginal mucosa and invading into the rectal wall	Surgery [3 months: uneventful recovery and ileostomy was closed]

TAH, total abdominal hysterectomy; SO, salpingo-oophorectomy; ERT, oestrogen-replacement therapy. Surgical menopause refers to procedures involving bilateral oophorectomy.

Sixteen other similar accounts were retrieved from our search, with the latest report published in 2009 (Giarenis *et al.*, 2009). The majority of cases (12 out of 17) were women with a prior hysterectomy, who took unopposed oestrogen. The remaining cases of recurrence were in women who took combined HRT.

#### Severity of prior endometriosis and menopause

Six patients had a history of ‘extensive’ or ‘severe’ endometriosis (Manyonda *et al.*, 1989; Joseph *et al.*, 1994; Taylor *et al.*, 1999; Badawy *et al.*, 2004). Fourteen patients underwent surgical menopause years before presentation (Skor *et al.*, 1977; Stewart and Ireland, 1977; Kapadia *et al.*, 1984; Ray *et al.*, 1985; Manyonda *et al.*, 1989; Joseph *et al.*, 1994; Taylor *et al.*, 1999, 2005; Badawy *et al.*, 2004; Giarenis *et al.*, 2009), while only two transitioned naturally to menopause (Goh and Hall, 1992; Chahine *et al.*, 2007). One patient entered menopause as a result of premature ovarian insufficiency (Mattar *et al.*, 2008). The median time between surgical menopause and presentation was 7.1 years (range: 4 months to 13 years).

#### Menopausal management

Unopposed oestrogen was implicated in numerous cases of symptom recurrence (*n* = 12) (Skor *et al.*, 1977; Stewart and Ireland, 1977; Kapadia *et al.*, 1984; Ray *et al.*, 1985; Manyonda *et al.*, 1989; Taylor *et al.*, 1999, 2005; Badawy *et al.*, 2004; Giarenis *et al.*, 2009). Fewer studies reported recurrence in women who were using combined hormonal preparations (oestrogen and progestagen) (*n* = 5) (Goh and Hall, 1992; Joseph *et al.*, 1994; Badawy *et al.*, 2004; Chahine *et al.*, 2007; Mattar *et al.*, 2008). In terms of method of oestrogen administration, oral tablets (*n* = 5) (Skor *et al.*, 1977; Stewart and Ireland, 1977; Kapadia *et al.*, 1984; Ray *et al.*, 1985; Goh and Hall, 1992), implants (*n* = 6) (Manyonda *et al.*, 1989; Taylor *et al.*, 1999, 2005) and patches (*n* = 3) (Badawy *et al.*, 2004; Taylor *et al.*, 2005) were all reported. Table III provides information on HRT dosages and regimens; however, variability in the level of detail provided by the included case reports and series limits these data.

**Table III:** HRT dosages and regimens associated with recurrent endometriosis in case reports and series.

HRT type	Dosages and regimens associated with recurrent postmenopausal endometriosis (*n* = 17 patients)
Oestrogen-only	1. Premarin 1.25 mg/day (Skor *et al.*, 1977)
	2. Premarin (Kapadia *et al.*, 1984)
	3. Oestrogens 2.5 mg/day (Ray *et al.*, 1985)
	4. 2.5 mg oestrogen/day, but taking twice the recommended dose (Stewart and Ireland, 1977)
	5. Oestrogen patch 0.05 mg twice a week (Badawy *et al.*, 2004)
	6. Conjugated oestrogen 1.25 mg/day (Giarenis *et al.,* 2009)
	7. Oestradiol implant 100 mg 6 monthly for 5 years, subsequent 2 years of taking no hormones, then 2 weeks before admission felt run down and took three 10 μg tablets of ethinyloestradiol on consecutive days (Manyonda *et al.*, 1989)
	8. 100 mg oestradiol annual hormone implants (Manyonda *et al.,* 1989)
	9. Oestradiol implant (Taylor *et al.*, 1999)
	10. Oestradiol implant (Taylor *et al.,* 1999)
	11. Oestradiol implant (Taylor *et al.*, 1999)
	12. First oestradiol implant, then Evorel 50 patches (Taylor *et al.,* 2005)
Combined	13. Conjugated equine oestrogens 625 mcg, norethisterone 150 mcg cyclically (Mattar *et al.,* 2008)
	14. Premarin 0.625 mg daily and Provera 10 mg daily for 12 days a month (Goh and Hall, 1992)
	15. Oestrogen + progesterone (Joseph *et al.*, 1994)
	16. Oestrogen patch 0.05 mg weekly followed by various regimens of oestrogen and progestogen (Badawy *et al.,* 2004)
	17. Oestradiol and cyproterone (Chahine *et al.*, 2007)

#### Presenting symptoms and sites of recurrence

As may be expected, endometriosis recurrence commonly presented with pain (*n* = 7): in locations typical of premenopausal endometriosis, i.e. abdomen (Taylor *et al.*, 1999), iliac fossae (Manyonda *et al.*, 1989; Goh and Hall, 1992), genitals (Taylor *et al.*, 1999); and in more unusual sites such as the loin (Manyonda *et al.*, 1989). Abnormal bleeding was also a common presentation (*n* = 14), including postmenopausal vaginal bleeding (Taylor *et al.*, 1999; Badawy *et al.*, 2004; Giarenis *et al.*, 2009), haematuria (Skor *et al.*, 1977; Stewart and Ireland, 1977; Kapadia *et al.*, 1984; Ray *et al.*, 1985; Taylor *et al.*, 2005), rectal bleeding (Taylor *et al.*, 2005) and also haemoptysis (Joseph *et al.*, 1994; Chahine *et al.*, 2007). Two women were reported to have pelvic masses (Skor *et al.*, 1977; Mattar *et al.*, 2008).

Sites of recurrence included: the genitourinary tract (*n* = 14), i.e. bladder (Skor *et al.*, 1977; Stewart and Ireland, 1977; Taylor *et al.*, 2005), ureter (Kapadia *et al.*, 1984; Ray *et al.*, 1985; Manyonda *et al.*, 1989; Taylor *et al.*, 1999; Giarenis *et al.*, 2009), ovary (Goh and Hall, 1992), cervix (Badawy *et al.*, 2004), vagina (Taylor *et al.*, 1999; Giarenis *et al.*, 2009); gastrointestinal organs (*n* = 4), i.e. the bowel (Stewart and Ireland, 1977; Taylor *et al.*, 2005; Mattar *et al.*, 2008) and rectum (Giarenis *et al.*, 2009); and the pulmonary system (*n* = 2) including the lung (Joseph *et al.*, 1994) and bronchus (Chahine *et al.*, 2007). Mattar *et al.* (2008) reported an unusual presentation involving an ovarian endometriotic cyst adherent to the small bowel with a solitary vascular pedicle (Mattar *et al.*, 2008). The authors hypothesized spillage during previous surgery and reactivation with hormonal therapy was responsible for this presentation.

#### Management of postmenopausal endometriosis recurrence

Management was tailored to location and extent of recurrence. All cases, except for one (Chahine *et al.*, 2007), required surgical excision of endometriotic tissue. Some patients required additional medical therapy involving Depo-Provera (Skor *et al.*, 1977), Danazol (Manyonda *et al.*, 1989) and Tibolone (Taylor *et al.*, 1999; Mattar *et al.*, 2008). Three patients resumed HRT after surgery (Goh and Hall, 1992; Taylor *et al.*, 1999; Badawy *et al.*, 2004), two of whom were prescribed oestrogen-only formulations (Goh and Hall, 1992; Badawy *et al.*, 2004).

Outcomes were generally favourable, although reporting bias may have contributed to this finding. All patients with reported follow-up (range: immediate postoperative checks to 2 years post treatment) experienced symptom regression with no relapses in the follow-up period (Skor *et al.*, 1977; Stewart and Ireland, 1977; Kapadia *et al.*, 1984; Manyonda *et al.*, 1989; Goh and Hall, 1992; Joseph *et al.*, 1994; Taylor *et al.*, 1999; Chahine *et al.*, 2007; Giarenis *et al.*, 2009).

### Malignant transformation (25 patients)

Twenty case reports and series of malignant transformation of endometriotic foci in postmenopausal women with a history of endometriosis on HRT were identified. This included a total of 25 patients between the ages of 38 and 75 years old (mean: 56 years) (Table IV). An additional study by Leiserowitz and colleagues (2003) (Leiserowitz *et al.*, 2003) detailing the malignant transformation of endometriosis in six postmenopausal women on oestrogen-only HRT (mean duration: 23.4 years) is included in Table IV, but will be discussed separately.

**Table IV:** Case reports and series reporting malignant transformation of endometriotic foci after HRT in women with a history of endometriosis.

Author, date (# patients)	Patient age (years)	Presenting symptoms [ureter involvement]	Medical history and menopause	HRT [duration]	Diagnosis	Treatment [follow-up: patient status]
Brooks and Wheeler (1977) (*N* = 1)	48	Mild right-sided lower abdominal pain, urinary frequency, constipation for 2 months [No]	Pelvic endometriosis, left ovarian endometriosis, adenomyosis, leiomyomas Surgical menopause	Oestrogen-only HRT Conjugated oestrogen tablets [4 years]	Clear cell carcinoma arising in endometriosis of the retroperitoneum	Surgery + radiation [22 months: no evidence of disease]
Klug *et al.* (1987) (*N* = 1)	66	Light vaginal bleeding [No]	Genital endometriosis Natural menopause + TAH/BSO 6 years before presentation	Oestrogen-only HRT Conjugated oestrogen tablets [12 years]	Endometrioid carcinoma arising in an endometriotic lesion of the cul-de-sac	Surgery [Unclear]
Reimnitz *et al.* (1988) (*N* = 2)	58 47	Vaginal bleeding and left flank pain [Yes] Nausea, vomiting, fever and flank pain [Yes]	(1) Extensive endometriosis and leiomyomata Surgical menopause (2) Extensive pelvic endometriosis Surgical menopause	Both oestrogen-only HRT Both conjugated oestrogen tablets [12 years] [4 years]	Extra-ovarian endometrioid carcinoma arising in foci of endometriosis Adenocarcinoma + adenosquamous carcinoma arising in foci of endometriosis 3 months later	Surgery + progestin therapy [60 months: no evidence of disease] Surgery + progestin therapy + radiation + chemotherapy [11 months: patient deceased]
Duun *et al.* (1993) (*N* = 1)	62	Pelvic mass [No]	Severe endometriosis (both ovaries and extensions deep into the rectovaginal septum) Surgical menopause	Oestrogen-only HRT Intramuscular oestrogen injections [20 years]	Endometrioid adenocarcioma (extraluminal rectosigmoid tumour 10 cm in diameter and closely adherent to the bladder)	Surgery + radiation [6 weeks: patient deceased]
Abu *et al.* (1997) (*N* = 1)	38	Intermittent vaginal bleeding of 8 weeks duration, ulcerated area over vaginal vault, polyp-like lesion on vaginal vault found 4 weeks later [No]	Severe endometriosis involving both ovaries, adenomyosis of the uterus, chronic cervicitis Surgical menopause	Oestrogen-only and combined HRT Ethinyloestradiol tablets [Not specified] Levonorgestrel/ethinyloestradiol tablets [Not specified] Oestradiol implants [Not specified]	Endometrial adenocarcinoma arising from an endometriotic focus	Neoadjuvant progestin therapy + surgery + radiation [Not specified]
Taylor *et al.* (1999) (*N* = 1)	42	Massive ascites, 6L drained [No]	Severe endometriosis Surgical menopause	Oestrogen-only HRT Oestradiol implants [7 years]	Endometroid adenocarcinoma	Surgery + chemotherapy [24 months: no evidence of disease]
Jimenez *et al.* (2000) (*N* = 1)	48	Right flank discomfort [Yes]	Endometriotic ovarian cyst Surgical menopause	Oestrogen-only HRT Conjugated oestrogen tablets [5 years]	Adenosquamous endometrioid carcinoma arising from disseminated pelvic endometriosis	Surgery [Not specified]
Powell *et al.* (2001) (*N* = 1)	56	Lower abdominal pain, dyspareunia, pain with bowel movements, hirsutism [Yes]	Extensive pelvic adhesions, peritoneal endometriosis, left ovarian endometrioma, adenomyosis, multiple leiomyomas Surgical menopause	Oestrogen-only HRT and combined HRT Conjugated oestrogen tablets [10 years] Medroxyprogesterone tablets [3 years]	Androgen-producing endometrioid tumour of low malignant potential (borderline tumour) arising in endometriosis in the rectovaginal septum	Surgery + chemotherapy + progestin therapy [9 months: no evidence of disease]
Jones *et al.* (2002) (*N* = 1)	52	Rectal bleeding and polyp arising from sigmoid colon [Yes]	Deeply infiltrating rectovaginal endometriosis Surgical menopause	Oestrogen-only HRT Oestradiol implants [12 years]	Well-differentiated endometrial adenocarcinoma arising from endometriosis of the rectosigmoid colon	Surgery [9 months: no evidence of disease]
Montamedi (2002) (*N* = 1)	57	Right lower-quadrant pain, recurrent macroscopic haematuria and weight loss of 8 kg in 3 months [Yes]	Uterine fibroids and right-sided endometrioma Surgical menopause (left ovary and fallopian tube left intact)	Oestrogen-only HRT Oestradiol [‘many years’]	Tubulopapillary endometrioid adenocarcinoma involving blood vessels	Surgery + radiation [18 months: no evidence of disease]
Petersen *et al.* (2002) (*N* = 2)	61 57	Diarrhoea, right buttock pain, rectal mass [No] Lower abdominal pain, rectal mass [No]	(1) Endometriosis Surgical menopause (2) Endometriotic foci on uterus, haemorrhagic endometriotic ovarian cyst Surgical menopause	Oestrogen-only HRT [5 years] HRT [8 years]	Poorly differentiated endometrioid adenocarcinoma of the large intestine arising in colorectal endometriosis Variably differentiated endometrioid adenocarcinoma	Surgery [28 months: no evidence of disease] Surgery [18 months: no evidence of disease]
Leiserowitz *et al.* (2003)^a^ (*N* = 10)	54.9 (mean)	Not reported	Not reported Surgical menopause (*n* = 8)	Oestrogen-only HRT (*n* = 6) [23.4 years (mean), 10–32 years (range)]	Endometrioid (*n* = 5) Adenosquamous (*n* = 2) Papillary adenocarcinoma (*n* = 1) Adenocarcinoma not otherwise specified (*n* = 2)	Surgery, chemotherapy, radiation [26 months (mean): 70% survival]
Soliman and Evans (2004) (*N* = 2)	60 51	Heavy, painless vaginal bleeding, palpable fixed mass, polypoid necrotic lesion [Yes] Painless mass in the right side of abdomen and several episodes of vaginal bleeding [Yes]	1) Pelvic endometriosis, microinvasive squamous carcinoma of the cervix, leiomyoma Menopause not specified (surgical menopause at age 57) 2) Extensive endometriosis involving the uterosacral ligaments, endometriotic right ovarian cyst Surgical menopause (left ovary and Fallopian tube left intact)	1) Oestrogen-only HRT Conjugated equine oestrogen tablets [3 years] 2) Combined HRT Oestrogen and testosterone implants [10 years]	Moderately differentiated endometrial adenocarcinoma Endometrioid adenocarcinoma arising in an endometriotic cyst within the vaginal vault	Surgery and radiation [48 months: no evidence of disease] Surgery [24 months: no further tumour growth]
Areia *et al.* (2004) (*N* = 1)	53	Abnormal vaginal bleeding for 2 months [Yes]	Leiomyomata, endometrioid foci especially in Fallopian tubes Surgical menopause	Oestrogen-only HRT Oestradiol [6 years]	Endometrioid adenocarcinoma affecting the vagina, bladder and rectum	Surgery + chemotherapy [6 months: no evidence of disease]
Kawate *et al.* (2005) (*N* = 1)	62	Abdominal mass [No]	Infiltrating pelvic endometriosis, leiomyoma Surgical menopause	Oestrogen-only HRT Conjugated oestrogen tablets [14 years]	Endometrioid adenocarcinoma arising from endometriosis of the mesocolon	Surgery + chemotherapy [28 months: no evidence of disease]
Noel *et al.* (2006) (*N* = 1)	75	Chronic abdominal pain for 2 months, left pyelonephritis [Yes]	Extensive endometriosis and adenomyosis Surgical menopause	Oestrogen-only HRT Super concentrated phytoestrogen supplements [5 years]	Ureteral malignant mullerian carcinosarcoma in a context of florid endometriosis	Surgery + aromatase inhibitor [3 months: no evidence of disease]
Milam *et al.* (2006) (*N* = 1)	47	Persistent and enlarging right groin mass, right lower-quadrant tenderness [No]	Recurrent endometriosis, left and right endometriomas Surgical menopause	Oestrogen-only HRT Oestrogen tablets [16 years]	Adenosarcoma arising in endometriosis	Surgery [12 months: no evidence of disease]
Al-Talib *et al.* (2008) (*N* = 1)	68	Left-sided pelvic mass, shortness of breath [No]	Endometriosis and multiple large fibroids Surgical menopause	Oestrogen-only HRT [13 years]	Metastatic endometrioid adenocarcinoma	Chemotherapy [24 months: patient is alive and well]
Chung *et al.* (2008) (*N* = 1)	66	Abdominal/pelvic pain and mass, alteration of general state, 10 kg weight loss, constipation, dysuria [Yes]	Never had endometriosis-specified pain, but unclear whether patient had endometriosis Natural menopause	Combined HRT Oestradiol and medroxyprogesterone acetate [11 years]	Extragenital endometrioid carcinoma in the vesico-uterine pouch arising from endometriosis	Surgery + chemotherapy + radiation [8 months: patient deceased]
Efthymiou (2009) (*N* = 1)	59	8-week history of constipation, tenesmus, 7 kg weight loss [No]	Severe ovarian endometriosis and in floor of pelvis Surgical menopause	Combined HRT Oestradiol and testosterone implant [13 years]	Well-demarcated, cystic, endometrioid adenocarcinoma (endometriosis-associated intestinal tumour)	Surgery [Not specified]
Karanjgaokar *et al.* (2009) (*N* = 3)	60 56 55	Intermittent right-sided abdominal pain for 4 years [Yes] Recent onset of discomfort in her right iliac fossa [No] Cramp-like discomfort in the abdomen and thighs and postmenopausal bleeding [No]	1) Ovarian endometriosis and adenomyosis Surgical menopause 2) Widespread endometriosis in the ovary, uterine serosa and bowel Surgical menopause 3) Endometriosis and left ovarian endometrioma Surgical menopause	1) Oestrogen-only HRT Oestrogen implants [14 years, discontinued 8 years before presentation] 2) Combined HRT Oestradiol and testosterone implants [12 years, discontinued 4 years before presentation] 3) Combined HRT Oestrogen and testosterone implants [9 years]	Adenosarcoma with heterologous leiomyosarcomatous element and moderately differentiated endometrioid adenocarcinoma Endometrioid adenocarcinoma High grade endometrial stromal sarcoma arising in residual foci of endometriosis and infiltrating the bowel	Surgery + aromatase inhibitor + chemotherapy Surgery + chemotherapy Surgery + chemotherapy[All cases were still being treated at the time of publication]

TAH/BSO, total abdominal hysterectomy and bilateral salpingo-oophorectomy.

^a^Article describes a total of 27 women with endometriosis-related malignancy; however, it is unclear how many of these women were postmenopausal. A subgroup of women with extra-ovarian disease (*n* = 10) included eight women with a history of hysterectomy and BSO, and one further woman who was taking unopposed oestrogens (therefore was presumably menopausal). For the purposes of this analysis, only these 10 women are described.

#### Severity of prior endometriosis

About 13 patients had medical histories that noted endometriosis in more than one site (Brooks and Wheeler, 1977; Duun *et al.*, 1993; Abu *et al.*, 1997; Powell *et al.*, 2001; Jones *et al.*, 2002; Petersen *et al.*, 2002; Soliman and Evans, 2004; Milam *et al.*, 2006; Noel *et al.*, 2006; Efthymiou, 2009; Karanjgaokar *et al.*, 2009), and 13 patients had histories of ovarian endometriosis (Brooks and Wheeler, 1977; Duun *et al.*, 1993; Abu *et al.*, 1997; Jimenez *et al.*, 2000; Powell *et al.*, 2001; Montamedi, 2002; Petersen *et al.*, 2002; Soliman and Evans, 2004; Milam *et al.*, 2006; Efthymiou, 2009; Karanjgaokar *et al.*, 2009). Some histories specified ‘severe endometriosis’ (*n* = 4) (Duun *et al.*, 1993; Abu *et al.*, 1997; Taylor *et al.*, 1999; Efthymiou, 2009), while others reported ‘extensive endometriosis’ (*n* = 4) (Reimnitz *et al.*, 1988; Soliman and Evans, 2004; Noel *et al.*, 2006). Some histories included comorbidities such as leiomyomas (*n* = 6) (Brooks and Wheeler, 1977; Reimnitz *et al.*, 1988; Powell *et al.*, 2001; Areia *et al.*, 2004; Soliman and Evans, 2004; Kawate *et al.*, 2005) and adenomyosis (*n* = 5) (Brooks and Wheeler, 1977; Abu *et al.*, 1997; Powell *et al.*, 2001; Noel *et al.*, 2006; Karanjgaokar *et al.*, 2009). Patients typically underwent surgical menopause (*n* = 22) (Brooks and Wheeler, 1977; Reimnitz *et al.*, 1988; Duun *et al.*, 1993; Abu *et al.*, 1997; Taylor *et al.*, 1999; Jimenez *et al.*, 2000; Powell *et al.*, 2001; Jones *et al.*, 2002; Montamedi, 2002; Petersen *et al.*, 2002; Areia *et al.*, 2004; Soliman and Evans, 2004; Kawate *et al.*, 2005; Milam *et al.*, 2006; Noel *et al.*, 2006; Al-Talib *et al.*, 2008; Efthymiou, 2009; Karanjgaokar *et al.*, 2009). Two additional patients underwent oophorectomy at ages 60 (Klug *et al.*, 1987) and 57 (Soliman and Evans, 2004) respectively; however, it was unclear in these two cases whether the patients had already naturally transitioned to menopause.

#### Menopausal hormonal preparations

HRT commonly consisted of unopposed oestrogens (*n* = 19) (Brooks and Wheeler, 1977; Klug *et al.*, 1987; Reimnitz *et al.*, 1988; Duun *et al.*, 1993; Abu *et al.*, 1997; Taylor *et al.*, 1999; Jimenez *et al.*, 2000; Powell *et al.*, 2001; Jones *et al.*, 2002; Montamedi, 2002; Petersen *et al.*, 2002; Areia *et al.*, 2004; Soliman and Evans, 2004; Kawate *et al.*, 2005; Milam *et al.*, 2006; Noel *et al.*, 2006; Al-Talib *et al.*, 2008; Karanjgaokar *et al.*, 2009) for a median duration of 6.7 years (range 3–20 years). Conjugated equine oestrogens (Premarin 1.25 mg/day or 0.625 mg/day) were frequently mentioned (*n* = 8) (Brooks and Wheeler, 1977; Klug *et al.*, 1987; Reimnitz *et al.*, 1988; Jimenez *et al.*, 2000; Powell *et al.*, 2001; Soliman and Evans, 2004; Kawate *et al.*, 2005). Oestradiol implants were also implicated (*n* = 4) (Abu *et al.*, 1997; Taylor *et al.*, 1999; Jones *et al.*, 2002; Karanjgaokar *et al.*, 2009), as well as oestrogen injections (*n* = 1) (Duun *et al.*, 1993). One interesting case of ureteral malignant mullerian carcinosarcoma in a 75-year-old woman was associated with 5 years of taking a phytoestrogen supplement (highly concentrated soy isoflavone) (Noel *et al.*, 2006).

Of note, two incidences of malignancy were reported in women who had discontinued HRT several years before presentation (Karanjgaokar *et al.*, 2009). In a case series of women over 55 years old, one woman used oestrogen implants (50–100 mg every 6 months) for 14 years post hysterectomy. She had stopped this regimen for 8 years before presenting with malignancies (adenosarcoma and endometrioid adenocarcinoma). Another patient in this series was on a regimen of 50 mg oestradiol and 50 mg testosterone implants for 12 years post surgical menopause. She was not on any form of HRT for 4 years before presenting with an endometrioid adenocarcinoma.

#### Presentation

Patients in these case reports/series presented with symptoms related to the site, extent, and type of malignancy. Vaginal bleeding was common (*n* = 7) (Klug *et al.*, 1987; Reimnitz *et al.*, 1988; Abu *et al.*, 1997; Areia *et al.*, 2004; Soliman and Evans, 2004; Karanjgaokar *et al.*, 2009; Suraweera *et al.*, 2012), as was pain in the abdomen/pelvis/buttock (*n* = 11) (Brooks and Wheeler, 1977; Powell *et al.*, 2001; Montamedi, 2002; Petersen *et al.*, 2002; Milam *et al.*, 2006; Noel *et al.*, 2006; Chung *et al.*, 2008; Karanjgaokar *et al.*, 2009). Masses were also frequently reported (*n* = 9) (Duun *et al.*, 1993; Petersen *et al.*, 2002; Soliman and Evans, 2004; Kawate *et al.*, 2005; Milam *et al.*, 2006; Al-Talib *et al.*, 2008; Chung *et al.*, 2008). Less frequent presentations included weight loss (*n* = 3) (Montamedi, 2002; Chung *et al.*, 2008; Efthymiou, 2009), constipation (*n* = 3) (Brooks and Wheeler, 1977; Chung *et al.*, 2008; Efthymiou, 2009) and flank pain (*n* = 3) (Reimnitz *et al.*, 1988; Jimenez *et al.*, 2000).

#### Malignant transformation and management

Malignant transformation of endometriotic foci was commonly diagnosed using Sampson's (Sampson, 1925) and Scott's (Scott, 1953) criteria. Endometrioid adenocarcinoma was by far the most commonly diagnosed HRT-associated malignancy in patients with a history of endometriosis (*n* = 18) (Klug *et al.*, 1987; Reimnitz *et al.*, 1988; Duun *et al.*, 1993; Abu *et al.*, 1997; Taylor *et al.*, 1999; Jones *et al.*, 2002; Montamedi, 2002; Petersen *et al.*, 2002; Areia *et al.*, 2004; Soliman and Evans, 2004; Kawate *et al.*, 2005; Al-Talib *et al.*, 2008; Chung *et al.*, 2008; Efthymiou, 2009; Karanjgaokar *et al.*, 2009). Other histological types included adenosarcoma (*n* = 2) (Milam *et al.*, 2006; Karanjgaokar *et al.*, 2009), clear cell carcinoma (*n* = 1) (Brooks and Wheeler, 1977), mullerian carcinosarcoma (*n* = 1) (Noel *et al.*, 2006), endometrial stromal sarcoma (*n* = 1) (Karanjgaokar *et al.*, 2009) and an androgen-producing endometrioid borderline tumour (*n* = 1) (Powell *et al.*, 2001). One interesting study reported an adenocarcinoma followed by an adenosquamous carcinoma arising in endometriotic foci 3 months later (Reimnitz *et al.*, 1988).

Treatments varied based on histological type, grade and stage of the tumour. In only one case it was decided to forego surgical management and treat solely with chemotherapy (Al-Talib *et al.*, 2008). In this case, the decision to initiate chemotherapy instead of surgery was based on her previous surgical history (two ileostomies) and poor prognosis due to advanced disease.

Adjuvant or neoadjuvant treatments in the form of chemotherapy (*n* = 9) (Reimnitz *et al.*, 1988; Taylor *et al.*, 1999; Powell *et al.*, 2001; Areia *et al.*, 2004; Kawate *et al.*, 2005; Chung *et al.*, 2008; Karanjgaokar *et al.*, 2009), radiation (*n* = 7) (Brooks and Wheeler, 1977; Reimnitz *et al.*, 1988; Duun *et al.*, 1993; Abu *et al.*, 1997; Montamedi, 2002; Soliman and Evans, 2004; Chung *et al.*, 2008) or progestin therapy (*n* = 4) (Reimnitz *et al.*, 1988; Abu *et al.*, 1997; Powell *et al.*, 2001) were frequently initiated. Mean follow-up was 19.4 months (range: 6 weeks to 5 years). Outcomes were generally favourable with no evidence of disease in 13 patients at follow-up (Brooks and Wheeler, 1977; Reimnitz *et al.*, 1988; Taylor *et al.*, 1999; Powell *et al.*, 2001; Jones *et al.*, 2002; Montamedi, 2002; Petersen *et al.*, 2002; Areia *et al.*, 2004; Soliman and Evans, 2004; Kawate *et al.*, 2005; Milam *et al.*, 2006; Noel *et al.*, 2006). The patient treated solely with chemotherapy was alive and well at 2 years after presentation (Al-Talib *et al.*, 2008).

#### Mortality

Although the majority of patients responded to treatment and were cured of their malignancy, three of the 25 patients diagnosed with an endometriosis-associated malignancy died as a result of their disease (Reimnitz *et al.*, 1988; Duun *et al.*, 1993; Chung *et al.*, 2008). Reimnitz *et al.* (1988) reported the case of a 47-year-old woman with a history of extensive pelvic endometriosis. She was on conjugated oestrogens (Premarin) 0.625 mg for 5 days every week for 4 years. She was initially diagnosed with a grade two adenocarcinoma arising from an endometriotic focus and obstructing the left ureter. Subsequently she was also diagnosed with moderately differentiated adenosquamous carcinoma arising from endometriotic foci. The patient was treated with cisplatinum and cyclophosphamide chemotherapy, but died after 11 months. In the case reported by Duun *et al.* (1993), the patient was a 62-year-old woman with a history of severe endometriosis involving both ovaries and the rectovaginal septum. She had received intramuscular oestrogen injections for 20 years following a hysterectomy and bilateral oophorectomy. After 3 years with no treatment, she resumed another hormone substitution regimen (not specified) for hot flushes. Within a year of commencing this hormonal substitution, the patient presented with a pelvic mass diagnosed as endometrioid adenocarcinoma. About 6 weeks after tumour excision, recurrence was diagnosed and the patient died despite radiotherapy. Chung *et al.* (2008) reported the case of a 66-year-old woman who presented with abdominal/pelvic pain and mass, alteration of general state, 10 kg weight loss, constipation and dysuria. She had used combined HRT (oestradiol and medroxyprogesterone acetate) for 11 years. After being diagnosed with extragenital endometrioid carcinoma in the vesico-uterine pouch arising from endometriosis, she was treated with surgery, chemotherapy and radiation. The patient was deceased 8 months later.

One unique case series by Leiserowitz *et al.* (2003) identified by our search reported on larger numbers of women and thus is presented separately. They describe the management of 27 women with endometriosis-related malignancy, identified during a 7-year period (by their presentation to one of the authors, and review of pathology records). The authors include all women with endometriosis-related malignancy, rather than exclusively postmenopausal women. However, it is clear from the article that a number of participants were postmenopausal. In particular, 10 women were identified with extragonadal (non-ovarian) malignancy, and 9 of these were clearly menopausal (with either a medical history of hysterectomy/BSO, or reported as using HRT). Of these 10 women, their malignancies were histologically described as endometrioid (*n* = 5), adenosquamous (*n* = 2), papillary adenocarcinoma (*n* = 1) or adenocarcinomas not otherwise specified (*n* = 2). Within this group, six women had taken unopposed oestrogen therapy for a mean duration of 23.4 years (range 10–32 years). The authors therefore suggest that unopposed oestrogen use could be a risk factor for endometriosis-associated malignancy, especially of non-ovarian location. Treatments included surgery, chemotherapy and radiation with a 70% reported survival at follow-up (mean: 26.3 months).

### Observational studies and clinical trials (6 studies)

Only six observational studies and clinical trials were identified by our search, highlighting the paucity of higher-level evidence in this area. These studies aimed to cover a variety of clinical questions, and the evidence for these is summarized below and in Table V. All assessed recurrence of endometriosis as their primary outcome.

**Table V:** Quality assessment of observational and clinical trials assessing risk of endometriosis recurrence after HRT.

Quality assessment							No of patients		Effect	Evidence quality
Studies	Study design	Risk of bias	Inconsistency	Indirectness	Imprecision	Other considerations	Intervention	Control	Relative (95% CI)	
**Comparison of HRT with no HRT**							**HRT**	**No treatment**		
Matorras *et al.* (2002)	Randomized trial	Serious^a^	Not serious	Not serious	Very serious^b^	None	4/115 (3.5%)	0/57 (0.0%)	**RR 4.50** (0.25 to 82.17)^c^	⨁◯◯◯ VERY LOW
Rattanachaiyanont *et al.* (2003)	Observational study	Very serious^d^	Not serious	Not serious	Very serious^b^	None	4/90 (4.4%)	0/17 (0.0%)	**RR 1.78** (0.10 to 31.64)^c^	⨁◯◯◯ VERY LOW
Acien *et al.* (2013)	Observational study	Serious^e^	Not serious	Not serious	Not applicable^f^	None	0/11 (0%)	0/8 (0%)	**Not calculable**^g^	⨁◯◯◯ VERY LOW
**Comparison of delayed HRT with immediate HRT**							**Delayed HRT**	**Immediate HRT**		
Hickman *et al.* (1998)	Observational study	Not serious	Not serious	Not serious	Not serious	None	(>6 weeks from surgery) 7/35 (20.0%)	(≤6 weeks from surgery) 4/60 (6.7%)	**HR 5.74** (1.31 to 25.23)^h^	⨁⨁◯◯ LOW
Arumugam and Damodaran (1998)	Observational study	Very serious^i^	Not serious	Not serious	Not applicable	None	(5 months from surgery) 0/8 (0%)	(3 months from surgery) 0/5 (0%)	**Not calculable**^g^	⨁◯◯◯ VERY LOW
**Comparison of HRT with tibolone**							**HRT**	**Tibolone**		
Fedele *et al.* (1999)	Randomized trial	Serious^j^	Not serious	Not serious	Very serious^b^	None	4/10 (40.0%)	1/11 (9.1%)	**RR 4.40** (0.59 to 33.07)^c^	⨁◯◯◯ VERY LOW
**Comparison of oestrogen-only HRT with combined HRT**							**Oestrogen-only HRT**	**Combined HRT (continuous and cyclical)**		
Rattanachaiyanont *et al.* (2003)	Observational study	Very serious^d^	Not serious	Not serious	Very serious^b^	None	4/50 (8.0%)	0/40 (0.0%)	**RR 7.24** (0.40 to 130.54)^c^	⨁◯◯◯ VERY LOW

CI, confidence interval; HR, hazard ratio; HRT, hormone replacement therapy; RR, risk ratio.

^a^High risk of performance bias—single blinded study, with physician unaware of treatment allocation, but with access to hormone results (which would have indicated treatment with HRT or not). High risk of detection bias, as assessment for recurrence was only carried out if the clinician felt this was warranted, which may have been influenced by the participant (who was not blind to treatment allocation).

^b^Very wide CI for RR.

^c^RR calculated by the authors using Review Manager (RevMan) [Computer program]. Version 5.3. Copenhagen: The Nordic Cochrane Centre, The Cochrane Collaboration, 2014.

^d^High risk of selection bias as unclear why women were allocated to different HRT regimens (or no HRT). High risk of detection bias, as researchers would have been aware of the woman's HRT status when assessing presence of recurrence (by reviewing medical records).

^e^Risk of detection bias, as criteria for designating recurrence are not clearly stated.

^f^Not applicable as odds ratio and CI cannot be calculated.

^g^No events in either group, therefore odds ratio not calculable.

^h^HR adjusted for stage of endometriosis, age at time of hysterectomy and postoperative adjunct medroxyprogesterone therapy.

^i^High risk of selection bias (unclear why some women started HRT after 3 months and some after 5 months), and high risk of detection bias (recurrence was only based on CA 125 levels).

^j^No description of blinding for the trial, and no scoring system is reported for pain, therefore risk of detection bias.

### Should HRT be given to women with previous endometriosis?

Given the concerns of possible disease reactivation or malignant transformation of endometriotic foci, it is reasonable to consider whether treatment with HRT is justifiable in this group of women. However, in a field dominated by case reports and series, it is challenging to obtain information on risk. Our search identified a single RCT and two cohort studies that give some insight regarding the risk of HRT in this cohort of women. All three studies were assessed as very low quality by GRADE criteria (Guyatt *et al.*, 2011).

The only RCT in this area was a single centre study from Spain, including a total of 172 women (Matorras *et al.*, 2002). All participants had a history of endometriosis and underwent BSO. Women were randomly allocated to treatment with combined HRT (50 μg oestradiol daily administered via patches and oral micronized progesterone for 14 days out of every 30 days) or no treatment. Participants were aware of their treatment allocation, although the clinician assessing them was not. In the treatment group, HRT was started 4 weeks following surgery. All women were followed up every 6 months with a clinical review, vaginal ultrasound and hormone measurements. Recurrence of endometriosis was identified either through histological confirmation, or by clinical findings (pelvic pain and/or pelvic mass) in association with pelvic ultrasound images suggestive of endometriosis. The overall absolute recurrence rate for endometriosis in this study was low at 2.3% (4/172). However, all women who experienced recurrence of endometriosis had been assigned to the HRT treatment arm (recurrence in 3.5% (4/115) of women compared to 0% (0/57) of women in the no treatment arm). The authors also suggested that the presence of residual endometrial tissue may be a possible risk factor for disease recurrence. In this cohort, the recurrence rate was 22.2% in women who had either a subtotal hysterectomy or BSO alone (2 out of 9 women). In contrast, the rate was only 1.9% (2 out of 106 women) in those who had total hysterectomy and BSO. The authors further suggest that a greater burden of disease may increase the risk of recurrence, as shown by an increased recurrence risk for women who had peritoneal involvement of greater than 3 cm, and a non-significant trend to increased recurrence with more advanced stages of endometriosis. The authors rightly noted that their study was underpowered to detect a statistically significant change in recurrence rates between the two groups of women. However, the study raises interesting possibilities for further research into the effect of disease stage and extent on recurrence rates.

One observational study included women who took postoperative HRT (of different regimens) and those who did not (Rattanachaiyanont *et al.*, 2003). In this retrospective, single centre cohort, the authors identified 107 women who had undergone hysterectomy and BSO for treatment of endometriosis. Women were treated with a variety of HRT regimens (total *n* = 90, taking unopposed oestrogen, continuous combined HRT, or cyclic HRT) or no treatment (*n* = 17). Recurrence was only identified in four women and all were receiving HRT, specifically unopposed oestrogen therapy. Three women had recurrent pain, and one woman had a vaginal nodule, confirmed as endometriosis on histology.

One further observational study reported on outcomes of women with deep infiltrating endometriosis and colorectal or rectovaginal disease, who underwent surgery without bowel resection (Acien *et al.*, 2013). This retrospective comparative cohort study was conducted in Spain and included women who were operated on at one of two hospitals. Of 42 patients, 19 had a hysterectomy and BSO, whilst the remainder had conservative surgery. Of the 19 women who underwent surgical menopause, 11 were subsequently treated with HRT, comprising 1–2 years of combined oestrogen/progesterone, followed by low dose oestrogen-only HRT or tibolone, continued indefinitely. The remaining 8 women did not receive HRT. The mean follow-up was 4.3 years (standard deviation 4.5, range 1–18). During this time, no women from either group were diagnosed with recurrence of endometriosis.

### Should HRT be given immediately following surgical menopause?

Further questions arise for women who undergo surgical menopause. If small deposits of endometriotic tissue remain following surgery, these may be triggered to proliferate by exogenous oestrogens given as HRT and increase the risk of recurrence or malignant transformation. Therefore, there may be a theoretical benefit in delaying the start of HRT, by allowing time for residual endometriotic tissue to regress before commencing exogenous oestrogen. Two retrieved articles, a retrospective cohort study (Hickman *et al.*, 1998) (GRADE: low quality) and non-comparative cohort (Arumugam and Damodaran, 1998) (GRADE: very low quality), attempted to investigate this question.

The retrospective cohort study (Hickman *et al.*, 1998) included women who underwent TAH with BSO, identified from the medical records of a single institution during a period of 12 years (1979–1991). Two groups of women were identified: those who commenced HRT within 6 weeks of their surgery (*n* = 60) and those who delayed starting HRT for at least 6 weeks (*n* = 35, mean time to starting HRT 71.1 weeks, range 7–520 weeks). Women who did not receive HRT were excluded. Information on symptom recurrence was obtained through the medical records or telephone follow-up, but a precise definition of recurrence was not reported. The mean duration of follow-up was 4.5 years. In unadjusted analyses, 4/60 (6.7%) women who began HRT immediately had recurrent pain, compared to 7/35 (20%) women who began HRT later on. Of note, in their adjusted analyses, in which endometriosis stage, age and postoperative adjunct medroxyprogesterone therapy were considered, starting oestrogen-replacement therapy (ERT) more than 6 weeks after surgical menopause had a hazard ratio of 5.7 for pain recurrence (95% CI; 1.3, 25.2). The authors therefore conclude that there is no increase in the risk of recurrence for women who commenced ERT immediately, as compared to those who delayed treatment.

A non-comparative cohort study (Arumugam and Damodaran, 1998) prospectively followed 13 women at one institution in Malaysia undergoing TAH and BSO for moderate or severe endometriosis. Patients were premenopausal at recruitment and had their endometriotic activity assessed by blood CA 125 levels taken pre-operatively and post-operatively (monthly). Eight patients received conjugated oestrogens in the form of Premarin (oral dose of 0.625 mg/day) starting 5 months post surgery, and five patients received oestrogens 3 months post surgery. Preoperative CA 125 levels were high in all 13 patients and declined to normal post surgery. Levels did not rise during the 6-month follow-up period and patients remained well and asymptomatic.

### What menopausal treatments are most appropriate for women with previous endometriosis?

If a woman with a history of endometriosis does decide to opt for HRT, then the next decision must be to choose the most suitable preparation. Again, there is limited high-quality evidence on which to base this decision. Two studies, retrieved by our search, provide some insight into this question. The first was a RCT (Fedele *et al.*, 1999) comparing HRT using transdermal oestradiol with tibolone, and the second was an observational study (Rattanachaiyanont *et al.*, 2003) comparing oestrogen-only HRT with combined HRT. Both were assessed as very low quality using the GRADE system.

The RCT (Fedele *et al.*, 1999) compared HRT (*n* = 10, transdermal oestradiol 50 mg twice weekly plus cyclic medroxyprogesterone acetate 10 mg daily for women with a uterus) and tibolone (*n* = 11, 2.5 mg orally once a day) in women with residual endometriosis after bilateral oophorectomy. Patients were randomized into one of the two treatment groups and followed for 1 year. Four patients in the oestradiol group experienced moderate pelvic pain during treatment compared to only one patient in the tibolone group. Furthermore, one patient in the HRT group discontinued treatment at 8 months due to the development of dyspareunia and post-coital bleeding from a vaginal mucosal endometriotic deposit. The authors concluded that tibolone may be a safer alternative for postmenopausal women with residual endometriosis, although note that their trial was very small.

The observational cohort study (Rattanachaiyanont *et al.*, 2003) attempted to compare various HRT regimens in women who had undergone hysterectomy and BSO for endometriosis. The majority of women (*n* = 50) were treated with unopposed oral oestrogen. Others were prescribed either continuous combined HRT (*n* = 24) or cyclical HRT (*n* = 16). Women received either conjugated equine oestrogens or oestradiol. Finally, a small group of women received no HRT (*n* = 17) and were viewed as controls. Women were followed up for a mean duration of 3.5 years (range 0.5–18 years). Although there were no statistically significant differences between the groups, the only episodes of recurrence (*n* = 4) were found in the oestrogen-only group. One woman who suffered with recurrent symptoms whilst taking oestrogen-only HRT also had relief of her symptoms when changing to a combined preparation. The authors conclude that HRT, particularly combined oestrogen and progestin regimens, is safe for postmenopausal women with underlying endometriosis.

## Discussion

In response to concerns regarding an increased risk of breast cancer raised by the Women's Health Initiative (Rossouw *et al.*, 2002) and the Million Women Study (Collaborators, 2003), HRT usage substantially decreased (Hersh *et al.*, 2004). Nevertheless, millions of women continue to rely on hormonal preparations for menopausal symptom relief and HRT remains the most effective treatment for menopausal vasomotor symptoms and vulvar and vaginal atrophy (Schmidt, 2012). However, amongst women with a history of endometriosis, HRT may entail additional risks, and to date there are no high-quality evidence-based guidelines to guide clinical decisions.

The articles described here provide insight into the management of menopausal symptoms amongst women with a prior history of endometriosis. The case reports and series included in our review, while limited in their usefulness in assessing prevalence, indicate that recurrence of endometriosis and malignant transformations can occur in postmenopausal women. Observational studies and clinical trials have further investigated the contingent risks of different forms and timing of HRT treatments.

### Recurrence

Endometriosis is not exclusively a premenopausal disease. About 13 case reports and series found 17 cases of recurrent endometriosis in postmenopausal women taking some form of HRT. These cases included women who underwent natural and surgical menopause; however, the vast majority underwent surgical menopause, perhaps indicating more severe premenopausal disease. Similarly, Vignali and colleagues (2005) reported substantial 5-year endometriosis recurrence rates, albeit amongst premenopausal women, of 43.5% (pain) and 28% (clinical disease) for women treated with conservative surgery (preservation of the uterus and at least one ovary) (Vignali *et al.*, 2005).

In the case reports, symptoms of postmenopausal endometriosis were similar to those reported in premenopausal endometriosis (Mounsey *et al.*, 2006): abnormal bleeding and pain. There are few papers describing the presentation of chronic pelvic pain (CPP) in postmenopausal women specifically. From clinical experience, symptoms are not cyclical unless a cyclical HRT preparation is used; however, pain associated with bowel and bladder function is common in postmenopausal women with CPP. Dyspareunia, especially superficially, may be associated with atrophic tissues but may also present in women using HRT with well-oestrogenized tissues. Deep dyspareunia, the more pathognomonic form of painful intercourse associated with endometriosis, is rarely described. Future research should investigate the prevalence of this symptom and whether and to what extent dyspareunia (superficial or deep) may contribute to postmenopausal sexual behaviour. Perhaps the most important difference in clinical practice, however, is the need to investigate new onset pelvic pain in a postmenopausal woman, whilst guidance on the management of CPP in reproductive age women emphasizes the need to avoid over-investigation and thus over-medicalization of the symptom when no underlying cause has previously been found (Home Page. Map of Medicine Web site. http://www.mapofmedicine.com/. [Accessed January 2017]). Findings from this review would suggest that investigation is needed when a postmenopausal woman is known to have had a previous diagnosis of endometriosis due to the added risk of malignant transformation.

The genitourinary system was the most common site of presentation, with many reports involving the ureter. This may represent the bias of case reports towards documenting more severe cases. Ureter involvement is a serious complication of endometriosis, capable of causing hydronephrosis and renal failure (Choi *et al.*, 2015). It has been suggested that most cases of ureteral endometriosis in postmenopausal women are actually a result of delayed presentation with onset prior to menopause (Yohannes, 2003). However, due to a lack of clinically relevant biomarkers and sufficiently specific imaging techniques, the onset of endometriosis remains unclear (May *et al.*, 2010; Dunselman *et al.*, 2014).

Prognosis was generally favourable after excision of endometriotic tissue. Randomized controlled trials corroborate these findings, reporting decreased pain and symptoms after laparoscopic surgery for endometriosis, although these trials did not specifically investigate ureteric surgery (Duffy *et al.*, 2014).

In three observational studies and one randomized clinical trial (Arumugam and Damodaran, 1998; Matorras *et al.*, 2002; Rattanachaiyanont *et al.*, 2003; Acien *et al.*, 2013), there appeared to be a small association between HRT and endometriosis recurrence, but there were no statistically significant differences between treatment and control groups. The current literature assessing risks of HRT in women with a history of endometriosis are uncertain, due to paucity of sufficiently large, high-quality studies. Current guidelines, consensus statements and recommendations acknowledge this deficit, but continue to emphasize the benefits of HRT over the undefined risks for severely symptomatic women (Al Kadri *et al.*, 2009; Johnson and Hummelshoj, 2013; Dunselman *et al.*, 2014). However, many women with endometriosis who undergo surgical menopause are given hormonal replacement therapy as a prophylaxis before the development of menopausal symptoms. For these women, clinicians must balance the benefits to bone (Cauley *et al.*, 2003) and cardiovascular health (Rossouw *et al.*, 2007), particularly for younger patients, against the potential risks of recurrence or malignancy.

It is important to note that recurrence is possible even in the absence of HRT. There are reports of endometriosis recurrence in women not on any hormonal treatment (Fujiu *et al.*, 2010; Bhat *et al.*, 2014). In these women, other risk factors such as hyperestrogenemia and obesity may play larger roles in the pathogenesis (Punnonen *et al.*, 1980). Incomplete definitive surgery and residual ovarian remnants are also considered risks factor for the development of postmenopausal endometriosis (Dmowski *et al.*, 1988). It remains to be confirmed whether a genetic predisposition together with environmental factors, medication, or fat distribution increase the risk of endometriosis after menopause, as has been shown for premenopausal women (Rahmioglu *et al.*, 2015a,b).

### Malignant transformation

Our search retrieved 20 case reports and series (25 patients) of malignant transformation of endometriotic foci following HRT. Of 25 patients, 22 had undergone surgical menopause, which was not surprising given that many women had a history of severe disease with comorbidities such as leiomyomas and adenomyosis. Unopposed oestrogens were implicated in 19 patients, with conjugated equine oestrogens implicated in eight patients and oestradiol implants in four patients. Currently there are no data to indicate the absolute risk of malignant transformation in this group of women. It is likely that this is a rare outcome, but better data are urgently needed to enable women to make an informed decision about menopausal management. Fortunately, tumours arising from endometriosis are typically low grade and have a better prognosis (Heaps *et al.*, 1990); only three deaths were reported in the literature identified by our search. Mortality was noted in the two case reports with patients who had histories of severe endometriosis and complicating factors, including increased age (Duun *et al.*, 1993) or multiple malignancies (Reimnitz *et al.*, 1988)

### Considerations regarding type and timing of HRT

Adjusting the type and timing of the treatment plan may mitigate the potential risks of HRT highlighted by our case reports and series.

#### Type: oestrogen-only, combined or tibolone

Our review retrieved evidence on three main types of HRT: oestrogen-only, combined and tibolone.

A consistent theme among the case reports is the predominance of oestrogen-only HRT in women with recurrence or malignancy. The majority of case reports concerned women taking unopposed oestrogens, particularly conjugated equine oestrogens. This is not surprising given the strong association between unopposed oestrogens and endometrial cancer (Sjogren *et al.*, 2016). As a result, current recommendations favour continuous combined preparations instead of unopposed oestrogens for women with a history of endometriosis, but the evidence remains sparse (Soliman and Hillard, 2006; Oxholm *et al.*, 2007). We identified a single observational study that addressed this issue, including only 90 women (Rattanachaiyanont *et al.*, 2003). Although the only women who developed recurrent symptoms were those taking oestrogen-only HRT, the study was retrospective and unable to demonstrate statistically significant differences between the groups. The authors suggested that combined HRT preparations might be the most appropriate for women with endometriosis who are using HRT. Large, randomized trials or observational studies with appropriate statistical power are clearly needed to clarify this question. Further research is urgently needed given the increased risk of breast cancer associated with combined HRT, although it is mostly in the older age group, which has been attributed to progestins (Chlebowski *et al.*, 2013).

Tibolone therapy has also been associated with recurrence of endometriosis (Sundar *et al.*, 2007). One RCT included in our review considered the use of tibolone, as compared with combined HRT, but the results should be interpreted with caution given the small sample size (*n* = 21). Fedele and colleagues (1999) concluded that tibolone (which typically has an oestrogenic effect on climacteric symptoms and bone, yet a progestogenic effect on tissues) might be a safer alternative to traditional HRT in patients with residual endometriotic disease, but no statistically significant difference was seen between the groups.

Notably, one case report highlights the importance of asking patients about their use of supplements or complementary/alternative medication. Five-year use of a highly concentrated isoflavone supplement was associated with florid recurrence of endometriosis and ureteral malignant mullerian carcinosarcoma (Noel *et al.*, 2006). This report raises further concerns over the use of phytoestrogens in postmenopausal women with a history of endometriosis (Cotroneo and Lamartiniere, 2001), despite some clinical and animal literature suggesting a reduced risk of endometriosis with dietary isoflavones (Tsuchiya *et al.*, 2007; Yavuz *et al.*, 2007). Given the high prevalence of supplement use, it is important to further explore the relationship between phytoestrogens and endometriosis.

#### Timing: initiation and duration

Data are also lacking on the optimal time to commence HRT following surgical menopause. We identified a retrospective study in this area, comparing immediate (within 6 weeks of surgery) to delayed (≥6 weeks following surgery) commencement of HRT (Hickman *et al.*, 1998). Although the crude incidence of recurrence was not different between the groups, increased recurrence was noted for women who delayed starting HRT after adjusting for confounders (AFS score at time of surgery, age at hysterectomy and postoperative adjunctive use of medroxyprogesterone). The authors themselves note the strong likelihood of bias in this observational study; it is probable that deferring the start of HRT would have been recommended to women felt to be at higher risk of recurrent symptoms. Additionally, we retrieved a non-comparative cohort study (Arumugam and Damodaran, 1998), which prospectively followed eight women who received conjugated oestrogens in the form of oral daily Premarin 5 months post surgical menopause, and five women who received oestrogens 3 months post surgical menopause. Women from both groups remained well and asymptomatic at 6-month follow-ups, yet clearly a much longer follow-up duration is necessary to be able to accurately assess risk of recurrence. The authors of this study also did not specify any symptoms or provide additional detail on patient status, and thus the evidence provided by this study was assessed as very low quality. Randomized trials are clearly needed to avoid this risk of bias, and have the potential to answer this question robustly.

Our search retrieved no studies investigating the total time for which women with histories of endometriosis should be treated. This is unfortunate given that it takes time to acquire mutations in endometriotic tissue, and thus duration of HRT therapy may have a large impact on probability of malignancy. Our systematic review also retrieved case reports of malignant transformation 4 and 8 years after stopping HRT treatment (Karanjgaokar *et al.*, 2009), indicating that hormone replacement may either still have effects years after discontinuation or that the use of HRT is only one factor in malignant transformation of endometriosis.

## Conclusion

**Figure 2:**
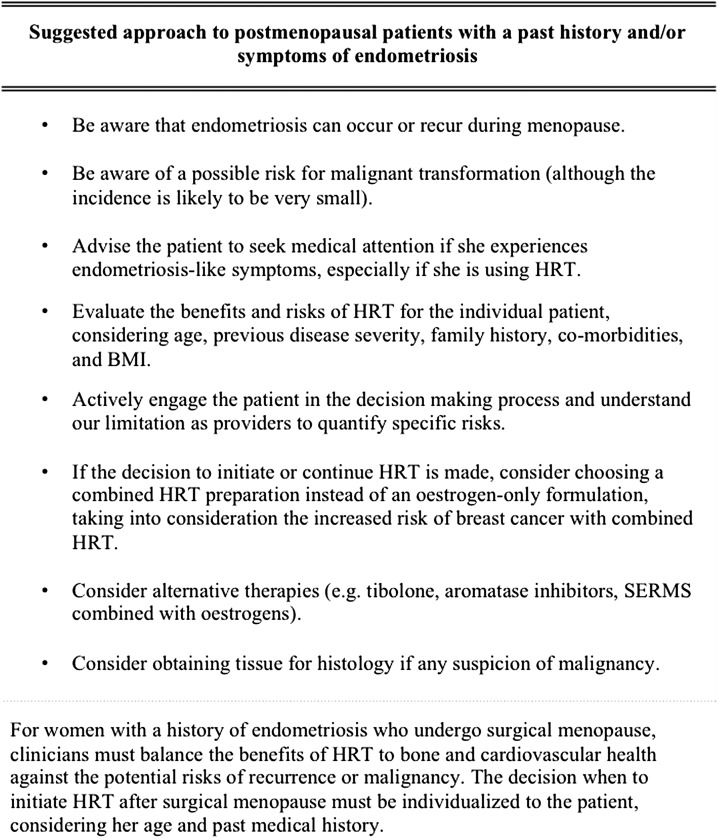
Suggested clinical approach to postmenopausal patients with a history of endometriosis.

Endometriosis is not exclusively a condition of the reproductive phase. Existing guidelines in this area emphasize the lack of evidence, but suggest that women should not be denied HRT treatment simply because of a history of endometriosis (Al Kadri *et al.*, 2009; Dunselman *et al.*, 2014). Our review indicates that women with a history of endometriosis should be carefully counselled about the possibility of disease recurrence after the menopause (Fig. 2). Although the absolute risk is unclear and likely to be low, women should be advised to seek help if they experience endometriosis-like symptoms, rather than suffer in silence. Furthermore, clinicians should adopt a cautious approach in cases of recurrence, keeping in mind the possibility of malignant transformation. For postmenopausal women with recurrent, treatment-resistant symptoms, consideration should be given to obtaining tissue for histology in order to exclude the possibility of malignancy, especially if other unusual or suspicious symptoms are present.

Although this review highlights potential risks of HRT, its substantial benefits should not be overlooked. In particular, the benefits may outweigh the costs of HRT for women with an early or surgical menopause. HRT has been shown to enhance cortical volumetric bone mineral density and compressive strength (Mikkola *et al.*, 2011; Kuh *et al.*, 2016). Additionally, studies have shown a reduced risk of coronary heart disease when hormonal therapy is administered to women with early natural or surgical menopause (Parker *et al.*, 2009). Importantly, unilateral or bilateral oophorectomy prior to the onset of natural menopause is associated with an increased risk of dementia and cognitive impairment (Rocca *et al.*, 2007); however, these risks may be reduced if HRT is administered up until the average age of natural menopause (Rocca *et al.*, 2014).

As with any woman commencing HRT, a full and frank discussion should be held about the risks and benefits of this treatment. Currently, clinicians must balance the benefits and risks of HRT, with attention to individual risk factors (age and BMI), and choose appropriate therapies directed at specific menopausal symptoms. Patients must be actively involved in the decision process, and understand our limitations as providers to quantify specific risks. Women should be advised that there are no robust data to indicate whether HRT changes the risk of disease recurrence or malignant transformation. Small studies have suggested the possibility of increased recurrence in women who take HRT, in particular unopposed oestrogen. Some authors advocate the use of combined HRT for women with a history of endometriosis, to minimize the risk of recurrence (Moen *et al.*, 2010; Dunselman *et al.*, 2014), but there are still risks with combined oestrogen-progestin hormone therapy. These include an elevated risk of breast cancer both during and post-intervention mostly in older women (Chlebowski *et al.*, 2015). Therefore care must be individualized, with the woman's personal and family history taken into account.

There are many promising areas for future research in this group of women. Our search retrieved no papers on the use of alternative selective oestrogen receptor modulators (SERMs) in postmenopausal patients with histories of endometriosis. We are aware of studies testing the gynaecologic safety of SERMS such as ospemifene and bazedoxifene, and combining these agents with oestrogens (especially bazedoxifene/conjugated oestrogens) (Mirkin *et al.*, 2016). Such studies have been promising, and may represent a future alternative to conventional HRT for our cohort.

## Summary

Our review highlights an important and severely under-researched area of gynaecology. The prevalence of endometriosis means that both specialists and general practitioners will inevitably encounter women with a history of this condition who are facing the dilemma of managing the menopause. Many women will have suffered years of debilitating symptoms before diagnosis, and then proceeded to undergo multiple treatments and operations in an attempt to regain some quality of life. These women deserve to have accurate, individualized and specific information about the risk of recurrence with different menopausal treatments, so that they can make an informed decision about their care.

## Supplementary Material

Supplementary Data
