# Supplementary material for: The management of menopause in women with a history of endometriosis: a systematic review
Source: Hum Reprod Update. 2017 May 11;23(4):481–500. doi: 10.1093/humupd/dmx011 (PMC5850813; doi:10.1093/humupd/dmx011)
Supplement: Supplementary Data [file suppl_table-1.docx]

**Supplementary Data**

MEDLINE search strategy

Database: MEDLINE(R) 1946 to June Week 3 2016

Platform: OVID

Search run on: 26 June 2016

1. Endometriosis/

2. endometrio$.ti,ab.

3. (extra adj1 endometrial).ti,ab.

4. Adenomyosis/

5. adenomyo$.ti,ab.

6. Endometrium/

7. (endometrial adj1 (hyperplasia or stimulation or proliferation)).ti,ab.

8. (stimulation adj2 endometrium).ti,ab.

9. Endometrial Hyperplasia/

10. or/1-9

11. Menopause/

12. Menopause, Premature/

13. Climacteric/

14. Postmenopause/

15. Primary ovarian insufficiency/

16. (cessation adj2 (menses or menstruation)).ti,ab.

17. (amenorrhea or amenorrhoea).ti,ab.

18. (postmenopaus$ or post-menopaus$).ti,ab.

19. (post adj1 menopaus$).ti,ab.

20. (menopaus$ or climacteric).ti,ab.

21. (premature adj1 menopause).ti,ab.

22. (primary adj1 ovar$ adj1 insufficiency).ti,ab.

23. (premature adj1 ovar$ adj1 failure).ti,ab.

24. (perimenopaus$ or peri-menopaus$).ti,ab.

25. (ovar$ adj1 follicle adj1 depletion).ti,ab.

26. hypoestrogenemia.ti,ab.

27. (final adj1 menstrual adj1 period).ti,ab.

28. Hysterectomy/

29. Hysterectomy, vaginal/

30. Ovariectomy/

31. (excision adj3 uterus).ti,ab.

32. (hysterectom$ or oophorectom$ or ovariectom$).ti,ab.

33. (total adj1 abdominal adj1 hysterectomy).ti,ab.

34. (female adj1 castration$).ti,ab.

35. (bilateral adj1 (ovariectom$ or oophorectom$)).ti,ab.

36. (bilateral adj1 salpingo-oophorectomy).ti,ab.

37. (pelvic adj1 radiation).ti,ab.

38. ((induced or medical or surgical) adj1 menopaus$).ti,ab.

39. (bilateral adj1 adnexectomy).ti,ab.

40. (hypo-estrogenism or hypoestrogenic or hypoestrogenism).ti,ab.

41. ((estrogen or oestrogen) adj1 deficiency).ti,ab.

42. (chronic adj1 gonadotropin-releasing adj1 hormone adj1 agonist adj1 therap$).ti,ab.

43. (GnRH adj1 agonist).ti,ab.

44. GnRH-a.ti,ab.

45. GnRH-analogue.ti,ab.

46. (pelvic adj1 clearance).ti,ab.

47. or/11-46

48. Hot flashes/

49. Sleep Apnea Syndromes/

50. Cognition Disorders/

51. Memory Disorders/

52. Cardiovascular Diseases/

53. Osteoarthritis/

54. Hyperlipidemias/

55. Skin Aging/

56. Sexual dysfunction, physiological/

57. Dyspareunia/

58. Mood disorders/

59. Depression/

60. Anxiety/

61. Fibromyalgia/

62. Arthralgia/

63. Myalgia/

64. Migraine disorders/

65. Dementia/

66. Osteoporosis, postmenopausal/

67. (night adj1 sweat$).ti,ab.

68. (hot adj1 (flash$ or flush$)).ti,ab.

69. (sleep adj1 apnea).ti,ab.

70. (cognitive adj1 change$).ti,ab.

71. (difficult$ adj1 concentrat$).ti,ab.

72. (memory adj1 loss).ti,ab.

73. (cardiovascular adj1 disease$).ti,ab.

74. (coronary adj1 heart adj1 disease).ti,ab.

75. (bone adj1 loss).ti,ab.

76. (vagina$ adj1 dryness).ti,ab.

77. osteoporosis.ti,ab.

78. (fracture$ or depression or anxiety or dementia or osteoarthritis).ti,ab.

79. (lipid adj1 profile).ti,ab.

80. (serum adj1 low-density adj1 lipoprotein).ti,ab.

81. (serum adj1 (HDL or LDL)).ti,ab.

82. (skin adj1 wrinkling).ti,ab.

83. (impaired adj1 balance).ti,ab.

84. dyspareunia.ti,ab.

85. (loss adj2 libido).ti,ab.

86. (sex$ adj1 dysfunction).ti,ab.

87. (stress adj1 incontinence).ti,ab.

88. (sleep adj1 disturbance$).ti,ab.

89. (mood adj1 symptom$).ti,ab.

90. ((joint or breast) adj1 pain).ti,ab.

91. (joint adj1 stiffness).ti,ab.

92. (breast adj1 tenderness).ti,ab.

93. (psychological adj1 symptom$).ti,ab.

94. (symptom adj1 recurrence).ti,ab.

95. (vasomotor adj1 symptom$).ti,ab.

96. (bone adj1 mineral adj1 density).ti,ab.

97. or/48-96

98. (managing or management or treatment$ or treating).ti,ab.

99. ((hormonal or non-hormonal or nonhormonal) adj1 therap$).ti,ab.

100. (regimen or medication or therapeutic).ti,ab.

101. or/98-100

102. Hormone replacement therapy/

103. Estrogen replacement therapy/

104. Estrogen/

105. Estradiol/

106. "Estrogens, Conjugated (USP)"/

107. "Estrogens, Esterified (USP)"/

108. Medroxyprogesterone Acetate/

109. progestogen.ti,ab.

110. Progesterone/

111. Progestins/

112. Selective Estrogen Receptor Modulators/

113. Progesterone Congeners/

114. Testosterone/

115. (menopaus$ adj1 hormone adj1 therap$).ti,ab.

116. MHT.ti,ab.

117. (hormon$ adj1 replacement adj1 therap$).ti,ab.

118. HRT.ti,ab.

119. (hormon$ adj1 (therap$ or replacement)).ti,ab.

120. (hormon$ adj1 add-back adj1 therap$).ti,ab.

121. (estrogen$ or oestrogen$ or estradiol).ti,ab.

122. (estrogen-based adj1 replacement adj1 therapy).ti,ab.

123. (unopposed adj1 estrogen-like adj1 stimulation).ti,ab.

124. Estrace.ti,ab.

125. (oral adj1 esterified adj1 (estrogen$ or oestrogen$)).ti,ab.

126. Menest.ti,ab.

127. (oral adj1 estropipate).ti,ab.

128. Ortho-est.ti,ab.

129. (oral adj1 conjugated adj1 equine adj1 (estrogen$ or oestrogen$)).ti,ab.

130. CEE.ti,ab.

131. Premarin.ti,ab.

132. (oral adj1 conjugated adj1 synthetic adj1 (estrogen$ or oestrogen$)).ti,ab.

133. (Cenestin or Enjuvia).ti,ab.

134. (oral adj1 estrogen-progestin adj1 combination$).ti,ab.

135. (Prempro or Prefest or Activella or Mimvey or FemHRT or Jinteli or Angeliq).ti,ab.

136. (estradiol adj1 patch$).ti,ab.

137. (Alora or Minivelle or Vivelle-dot or Climara or Menostar).ti,ab.

138. ((estrogen-progestin or oestrogen-progestin) adj1 patch$).ti,ab.

139. (Combi-Patch or Climara Pro).ti,ab.

140. ((estrogen or oestrogen) adj1 (gel or gels)).ti,ab.

141. (EstroGel or Elestrin or Divigel).ti,ab.

142. (estradiol adj1 emulsion).ti,ab.

143. Estrasorb.ti,ab.

144. (estradiol adj1 spray).ti,ab.

145. EvaMist.ti,ab.

146. (estradiol adj1 (intravaginal or vaginal or ring)).ti,ab.

147. (Femring or Estring).ti,ab.

148. (estradiol adj1 vaginal adj1 tablet).ti,ab.

149. Vagifem.ti,ab.

150. (estradiol adj1 vaginal adj1 cream).ti,ab.

151. (systemic adj1 (estrogen$ or oestrogen$)).ti,ab.

152. (medroxyprogesterone adj1 acetate).ti,ab.

153. (progesterone or MPA).ti,ab.

154. (vaginal adj1 progesterone).ti,ab.

155. (oral adj1 micronized adj1 progesterone).ti,ab.

156. (conjugated adj1 (estrogen-bazedoxifene or oestrogen-bazedoxifene)).ti,ab.

157. (oral adj1 conjugated adj1 equine adj1 (estrogen$ or oestrogen$) adj1 bazedoxifene).ti,ab.

158. Duavee.ti,ab.

159. (selective adj1 (estrogen$ or oestrogen$) adj1 receptor adj1 modulator$).ti,ab.

160. (bazedoxifene or SERM or Tibolone).ti,ab.

161. (ethinyl adj1 estradiol).ti,ab.

162. (bioidentical adj1 hormone adj1 therap$).ti,ab.

163. (testosterone adj1 (replacement or therap$)).ti,ab.

164. (androgen adj1 replacement).ti,ab.

165. (esterified adj1 estrogen-methyl adj1 testosterone).ti,ab.

166. (EEMT adj1 combination$).ti,ab.

167. hyperestrogenism.ti,ab.

168. (ultralow-dose adj1 unopposed adj1 estradiol).ti,ab.

169. gabapentin.ti,ab.

170. or/102-169

171. (isoflavone$ or phytoestrogen$ or phyto-estrogen$).ti,ab.

172. Phytoestrogens/

173. Isoflavones/

174. Soy Foods/

175. Plant Extracts/

176. Acupuncture/

177. Vitamin E/

178. Dietary Supplements/

179. Antidepressive Agents/

180. Serotonin Uptake Inhibitor/

181. (isoflavone$ or phytoestrogen$ or phyto-estrogen$).ti,ab.

182. (Paroxetine or Brisdelle or Paxil or Gabapentin or Neurontin or Venlafaxine or Effexor or Desvenlafaxine or Pristiq).ti,ab.

183. (Citalopram or Celexa or Escitalopram or Lexapro or Fluoxetine or Prozac or Sertraline or Zoloft or Clonidine or Catapres).ti,ab.

184. (plant-derived adj1 (estrogen$ or oestrogen$)).ti,ab.

185. (plant adj1 (estrogen$ or oestrogen$)).ti,ab.

186. (herbal adj1 treatment$).ti,ab.

187. (black adj1 cohosh).ti,ab.

188. (evening adj1 primrose adj1 oil).ti,ab.

189. (soy or acupuncture or antidepressant$ or SSRI or bisphosphonates).ti,ab.

190. (vitamin adj1 E).ti,ab.

191. (lifestyle adj1 (change$ or modification$)).ti,ab.

192. (dietary adj1 supplement$).ti,ab.

193. (selective adj1 serotonin adj1 reuptake adj1 inhibitor$).ti,ab.

194. or/171-193

195. 97 or 101 or 170 or 194

196. 10 and 47 and 195
